# Best Practices for Financial Sustainability of Healthy Food Service Guidelines in Hospital Cafeterias

**DOI:** 10.5888/pcd15.170477

**Published:** 2018-05-17

**Authors:** Stephanie Jilcott Pitts, Brittany Schwartz, John Graham, Amy Lowry Warnock, Angelo Mojica, Erin Marziale, Diane Harris

**Affiliations:** 1 East Carolina University, Greenville, North Carolina; 2 Gillings School of Global Public Health, University of North Carolina at Chapel Hill, Chapel Hill, North Carolina; 3 Division of Nutrition, Physical Activity and Obesity, Centers for Disease Control and Prevention, Atlanta, Georgia; 4 University of North Carolina at Chapel Hill, Chapel Hill, North Carolina; 5 National Network of Public Health Institutes, New Orleans, Louisiana

## Abstract

In February and March 2017 we examined barriers and facilitators to financial sustainability of healthy food service guidelines and synthesized best practices for financial sustainability in retail operations. We conducted qualitative, in-depth interviews with 8 hospital food service directors to learn more about barriers and facilitators to financial sustainability of healthy food service guidelines in retail food service operations. Analysts organized themes around headers in the interview guide and also made note of emerging themes not in the original guide. They used the code occurrence and co-occurrence features in Dedoose version 7.0.23 (SocioCultural Research Consultants) independently to analyze patterns across the interviews and to pull illustrative quotes for analysis. Two overarching themes emerged, related to 1) the demand for and sales of healthy foods and beverages, and 2) the production and supply of healthy foods and beverages. Our study provides insights into how hospital food service directors can maximize revenue and remain financially viable while selling healthier options in on-site dining facilities.

## The Challenge of Implementing and Sustaining Healthy Food Service Guidelines

Given the rising prevalence of obesity and associated health care costs (1–3), food service operations in the health care industry are under growing pressure to offer nutritious foods and beverages to customers at onsite dining facilities. With industry trends like the Partnership for a Healthier America’s (PHA’s) Healthy Hospital Food Initiative (4) and Health Care Without Harm's Healthy Food in Health Care Pledge (5), food service operations in the health care industry are committing to replacing calorie-dense and nutrient-poor foods and beverages with healthier options. However, there are several barriers to implementing such healthy food service guidelines, including customer complaints and dissatisfaction, need for increased labor skills, increased time needed to prepare healthier food, and inadequate selections offered from vendors (6).

Changes in current food practices will continue to occur as directors are tasked with balancing increased organizational pressure to generate a profit with pressure from the health care industry and the public to provide healthier food options. Little is known about how these healthy food service guidelines in retail operations have changed long-term revenue for hospitals and food service contractors. As healthy food service guidelines proliferate, more research is needed to understand best practices to improve financial sustainability once the guidelines are implemented. Thus, the purpose of this study was to examine barriers and facilitators to maintaining financial sustainability of healthy food service guidelines and to synthesize information on best practices for financial sustainability collected from 8 hospital food service directors. This information can help food service directors successfully transition to implementation of healthy food service guidelines in their retail operations in a financially sustainable way.

## The Search for Solutions

**Participant recruitment** *.* We used purposive sampling to recruit respondents. Participants were recruited by using the listserv of the Association for Healthcare Foodservice, the PHA web page, and personal contacts. Eligibility was limited to food service directors of sites that had implemented healthy food service guidelines in their onsite retail dining facility for at least 6 months. Each site was asked to classify its food service as self-operated, contracted, blended, or neither. Self-operated sites use internal funds, staff, and equipment to manage food service, whereas contracted food service is managed by an external vendor hired to handle all aspects of food service for the hospital dining facility. Of the 8 participating sites, one used a blended model and classified itself as both self-operated and contracted, 6 reported a self-operated model, and one classified itself as neither. 

**Data collection.** In-depth interviews were conducted in February and March 2017. Before the 8 key informant interviews, participants were asked to complete a brief Qualtrics survey about characteristics of their hospital food service operation (eg, number of beds, number of employees, group purchasing organization [GPO] used, broadline distributor used). In a GPO, numerous hospitals or other entities join together to pool purchasing power and obtain reduced pricing and rebates from manufacturers. A broadline distributor is a company that provides food and nonfood products to hospitals and functions as an intermediary between manufacturers and the hospital food service operator.

One-hour semi-structured interviews were conducted with pairs of interviewees with similar operational characteristics, to encourage information sharing. The same interviewer (S.J.P.) facilitated each interview. The research project was submitted to the Office of Human Research Ethics at the University of North Carolina at Chapel Hill, which determined that the submission did not constitute human subject research and did not require institutional review board approval.

**Interview guide.** A preliminary review of literature addressing financial sustainability for hospital cafeterias implementing healthy food service guidelines was conducted to inform development of the interview guide. S.J.P. developed an initial interview guide based on literature review results with contributions from all authors. Interview themes were 1) employee training/education, 2) menu-planning and recipe redevelopment, 3) procurement, 4) inventory and tracking systems, and 5) behavioral design strategies. Revisions to the guide were based on discussions among research team members and a pilot test with one food service director identified through purposive sampling. The interview guide was refined and finalized with 7 sections and 25 open-ended questions.

**Data analysis.** Interviews were transcribed verbatim using NoNotes transcription service (Nonotes.com). Researchers independently cleaned the transcripts for typographical errors before uploading into Dedoose (Dedoose.com), a collaborative web-based qualitative software system for health and policy research. S.J.P. and B.S. used 2 transcripts to develop independent codebooks and then developed a consolidated codebook with prioritized topical codes. All interview transcripts were cloned and independently coded by S.J.P. and B.S. using the consolidated codebook. S.J.P. and B.S. discussed the independently coded transcripts and resolved discrepancies in coding. Final coding decisions were documented in a single transcript.

S.J.P. and B.S. used the headers in the interview guide to organize themes and made note of emerging themes not included in the original interview guide. They independently used the code occurrence and co-occurrence features in Dedoose to analyze patterns across the interviews and extract illustrative quotes. The 2 independent documents listing themes were consolidated by the analysts. A list of potential best practices for financial sustainability for hospital food retail operations was collated as authors discussed each theme. A third analyst (J.G.) reviewed all themes, and further consolidated themes into best practices to maintain financial sustainability when implementing healthy food service guidelines. Participant checking was also used, wherein each food service director reviewed results and provided feedback. We describe characteristics of respondents’ hospital food service retail operations, followed by overarching themes and best practices related to 1) the demand for and sales of healthy foods and beverages, and 2) the production and supply of healthy foods and beverages.

The 8 participants worked in hospitals that ranged in size from 92 to 2000 beds, and 65 to 25,000 employees. The time required to implement healthy food service guidelines ranged from 6 months to 3 years or longer. Table 1 shows hospital cafeteria characteristics for each respondent. Two overarching themes emerged as necessary to ensure that hospital dining facilities remain financially sustainable when implementing healthy food service guidelines, related to 1) the demand for and sales of healthy foods and beverages, and 2) the production and supply of healthy foods and beverages (Table 2).

**Table 1:** Characteristics of Hospital Cafeterias Reported by Food Service Directors (N = 8), Study of Best Practices for Implementing and Sustaining Healthy Food Service Guidelines, 2017

Respondent Identifier	Geographic Location	Number of Hospital Beds	Number of Cafeteria Employees	Payment Model (Self-Operated or Contracted)	Group Purchasing Organization^a^	Broadline Distributor^b^
P01	West	176	1,200	Self-operated	Entegra	Sysco
P02	Midwest	1,400	14,000	Self-operated	Vizient	US Foods
P03	West	661	7,015	Self-operated	Vizient	US Foods
P04	Midwest	207	2,600	Self-operated	Vizient and Intalare	Gordon Food Service
P05	Midwest	2,000	12,000	Profit	Premier	US Foods
P06	Northeast	1,200	25,000	Self-operated	Premier	US Foods
P07	Northeast	401	Not provided	Self-operated	Premier	Gordon Food Service
P08	West	92	4,800	Neither	Not applicable	US Foods

a Numerous hospitals or other entities joined together to pool purchasing power to obtain reduced pricing and rebates from manufacturers.

b A company that provides food and nonfood products to hospitals and functions as an intermediary between manufacturers and the hospital food service operator.

**Table 2:** Illustrative Quotes From Food Service Directors (N = 8), Study of Best Practices for Implementing and Sustaining Healthy Food Service Guidelines, 2017

Best Practices	Illustrative Quotes^a^
**Theme: Demand for and sales of healthy foods and beverages**	
Evaluate new dishes with staff and customers to see which are received well.	P05: “I think we've had the most amount of success when we built up some excitement around taste-testing. Our dietitians get involved in soliciting and providing information and showing the consumers that many of the healthy items can be tasty and satisfying.”
Use point of sale (POS) and nutrition analysis software to identify and prioritize popular recipes that meet healthy food guidelines.	P04: “We use what we call CBORD^b^ and all of our recipes are put in there and that’s how we do the nutrition analysis to make sure that meals meet the criteria that we set for sodium and calories and saturated fats.” P06: “We have a POS system that we track sales through to look at our top-selling items. We can run many different reports to see what’s selling or what’s not selling.”
Anticipate dropping popular comfort foods by preparing customers in advance.	P07: “We removed the deep fryers, and prior to that there were quite a few items that were deep fried. We started this and communicated the changes a month in advance to the launch . . . Fried chicken was a very popular item, but we replaced it with baked chicken made with a pecan crust and we let the dining guests taste it . . . ”
Avoid setting healthy foods apart as something different; rather, attempt to improve the healthfulness of most dishes served at the cafeteria.	P07: “Our goal was not to say ‘healthy’ or ‘unhealthy’, but to make the entire menu healthier, and by posting nutritional information, people could make a choice . . . They call this approach ‘stealth health’, or the idea that you avoid labeling foods ‘healthy’ because if a food is labeled ‘healthy’, people will not want to eat it.”
To increase customer volume, roll out and launch new menus that feature tastier, healthy menu options.	P07: “. . . by creating a healthier menu, we actually created an entirely new market. We brought them in, we brought all the regular dining guests in and for the last 4 years we have consistently increased sales.”
Offset higher food costs for new healthier menu items by adjusting the price point of other popular food offerings to ensure overall profitability.	P03: “You know, it’s about a balance. I put in a healthy item and maybe instead of my 45% food cost that I want to have on it, its 55%. Then I offset that with something else being a little more costly and a little lower food cost and hopefully it’s something that’s less healthy.”
Realign employee and customer discounts to favor healthy menu options.	P04: “We decided to give consumers the choice and so we went from a system of a 20% discount for all food to a 25% discount for healthy food.”
**Theme: Production and supply of healthy foods and beverages**	
Regularly emphasize and share the goals of the healthy food program with staff, emphasizing the benefits of a healthier diet.	P03: “I think it’s another good reason to share what all the goals are and to make sure that the food service staff understands that it’s what the [healthy food service guideline program] is trying to get us to do.”
Instill pride in the production of better tasting, healthier foods, thereby enhancing staff productivity and retention.	P03: “Once you have people trained and once people buy into the program, [they] take pride in the fact that when I make my soup, we boil vegetables for a vegetable broth, we use all fresh ingredients that we chop up ourselves . . . People [food service staff] bought into that and became more productive because they liked what they were doing.”
Conduct taste tests with frontline staff so they buy in to healthy options and will then market them to consumers.	P07: “We got buy-in for our [implementation] changes from not only the employees, but our customers and our dining guests.”
Develop recipes that balance the cost of ingredients, but overall provide a healthy, delicious alternative.	P08: “Salmon is very expensive to sell and we could not sell it without losing money. So, to provide a healthier option, we chose to take the salmon filets and use them to make salmon croquettes. By adding some filler to the cooked salmon, we were able to bring the price point down, extending the salmon while keeping a great tasting, healthy product.”
Absorb losses associated with more expensive, healthier foods by offsetting higher costs with increases in the price of other popular dishes on the cafeteria’s larger menu.	P03: “We use an all-natural antibiotic-free chicken that is never frozen, and it’s a fabulous product. It costs more than any other chicken out there almost, but it’s what we chose quality-wise as what our facility wanted to do.”
Serve smaller portions of those foods that are more expensive to make.	P06: “For example, for national nutrition month this March, we did new parfaits. We were selling a 9-ounce parfait cup with pudding and cookies for $1.75. For March, we pulled all those items and we replaced them with a 7-ounce smaller portioned parfait that had whole grains, quinoa, fresh berries, and Greek yogurt and priced it at $2.50. We really didn't think that people would buy them, but they're the hottest seller right now.”
Introduce innovative training options that maximize the reach and impact of trainers.	P06: “So we have a demo kitchen that can tape-record live training sessions . . . producing training videos . . . that are accessible to all of our frontline food service employees.”
Adopt innovative technologies and obtain kitchen equipment to enhance labor efficiency and productivity.	P01: “We have some really nice Bain-maries and Planchas and other great equipment that we can use to decrease the oil and still make foods taste good.”
Work with your GPO to negotiate prices and secure reasonably priced ingredients for healthy foods.	P07: “ [Respondent’s GPO] is the one that negotiates the pricelist. I sat on the [respondent’s GPO] food service committee for 4 years . . . We actually had monthly meetings as far as negotiating contracts and what the needs were of the operations that were part of [respondent’s GPO].”
When managing multiple sites or facilities, use POS systems to determine top-sellers and to simplify inventory management.	P06: “One of the things we’ve done . . . is look at all of the salad dressings we have and ask, “Why do we need 17 different salad dressings?” We identified 7 salad dressings that are approved to use in the retail operation and the manager can scale up or down based on the size of the operation. We were able to really consolidate our inventory and our purchasing and save money that way.”
Identify and work with small, local vendors to achieve healthy food goals.	P07: “We sat down with our vendors and said we needed them to make a recipe that cuts sodium content in 10 breads in half. They did it. Within 3 weeks we had a new recipe and we had healthier items.”
Set up healthy stations to ensure portion control and distinguish healthy food choices.	P08: “For the salad bar, we have red, yellow, and green handled tongs and scoops. So instead of having little print cards out on the salad bar, we just had one sign in front of the salad bar and then we have the color coded handles there.”
Adopt behavioral design strategies to encourage healthy food selection and sales.	P01: “[W]e have a red/yellow/green for sugar-sweetened beverages so we give them a green for your waters and unsweetened teas, and then red is for the high sugar items, and then yellow is for, like, so it has a little bit of sugar but not to the degree that regular soda would have in it.”

Abbreviation: GPO, group purchasing organization.

a Eight food service directors participated in our interviews. P0x numbers identify each respondent (Table 1).

b CBORD is a diet/menu analysis system.

## Overarching Themes Related to Demand for and Sales of Healthy Foods and Beverages

From the demand and sales perspective, all 8 food service directors voiced concerns that the higher prices of healthy foods and highly restrictive healthy food service guidelines could reduce sales and overall profitability. All 8 food service directors noted that healthier foods are typically more expensive to purchase and prepare, and thus must be prepared and marketed creatively to ensure continued profitability. Five directors noted that customers were willing to accept smaller portions of tastier dishes or pay more for healthier products, like antibiotic-free or organic chicken. Although directors often could not state that profitability had increased because of healthier food selections, 5 noted that customer volume had increased or stayed the same. Six directors believed that, in some cases, it was workable to price healthy dishes at a loss to promote greater customer volume and to promote healthy food purchases.

A common concern voiced by 5 directors was that a substantial portion of the existing customer base could easily be alienated, with a commensurate reduction in sales, if the healthy food program was too aggressive and restrictive. “I was not a fan of that [healthy food service guidelines] program, I thought it was so restrictive . . . the reality is that if you tell somebody that they can’t have something, guess what — they want it even more.” (Participant [P]01)

One food service director employed a strategy called “stealth health” to ensure that customers were not turned off by the idea of overtly healthier options. Six said that it was challenging to drop comfort foods (eg, fried foods and sugar-sweetened beverages) and familiar, traditional foods from the menu because customers resisted these changes. Furthermore, the hospital setting can be stressful and customers often want comfort foods and beverages.

I’m amazed at how passionate customers have been about the fries. We took out the fries, and we still hear about it to this day and it’s been over 2 years ago. That’s why I didn’t take out the sugar-sweetened beverages because I didn’t want to hear about that. People are in the hospital and they are stressed and they need something that they consider comfort food, so I don’t want to deny that to people if that’s what makes them feel better. (P01)

## Best Practices for Increasing Demand for and Sales of Healthy Foods and Beverages

The following best practices were identified to address concerns about demand for and sales of healthy foods:

1. Evaluate new dishes with staff and customers to see which are well-received. For example, do successive taste-tasting before the roll-out of new dishes.
2. Use point of sale (POS) and nutrition analysis software to identify and prioritize popular recipes that meet healthy food service guidelines.
3. Anticipate dropping popular comfort foods by preparing customers in advance, and replace them with tasty, healthier alternatives.
4. Avoid setting healthy foods apart as something different; rather, attempt to improve the healthfulness of most dishes, and support customers’ ability to make healthy choices through food labeling.
5. To increase customer volume, roll out and launch new menus that feature tastier, healthy menu options. The goal should be to enhance the overall appeal of the menu and draw in new customers.
6. Offset higher food costs for new, healthier menu items by adjusting the price point of other popular food offerings to ensure overall profitability.
7. Realign employee and customer discounts to favor healthier menu options.

## Overarching Themes Related to Production and Supply of Healthy Foods and Beverages

From a supply perspective, 8 food service directors said they struggle with the cost of healthy food production and the effective engagement of their food service staff to embrace the organizational culture shift to healthier eating. All 8 noted that a limited supply of healthy packaged foods and ingredients is available from GPOs and broadline distributors. This makes it more expensive to prepare healthy foods because they cost more to develop, require more staff time to produce, and the recipe ingredients are more costly. “We found that some of our healthier recipes that are popular are a little bit more labor intensive. We really had to take a look at some of our labor and how we did our work.” (P05)

The 8 food service directors reported that, in addition to staff time associated directly with food production, a successful healthy food program requires staff training around recipe redevelopment and production, the display and promotion of healthy dishes, and the goals and practices of offering a healthy food program. One group had a particularly innovative training method wherein the executive chef provided videos for other employees, and disseminated the training online.

It's been a real big change for us and one of our initiatives from our executive chef since he started to do online trainings. We have a demo kitchen that can record live training sessions. He teaches people how to cook whole grains, how to rehydrate dried beans, all of these new ingredients that we're using that we haven't used in the past . . . he's doing training videos that are accessible to all of our frontline food service employees. (P06)

All 8 food service directors said that healthy food programs may require upfront capital investment to address both the cost of healthy food production and the availability of healthy food choices. Food service directors must determine what investments should be made to obtain kitchen equipment needed for healthy food production, and how to reduce labor costs associated with healthier food preparation and service.

Six food service directors expressed concern with the financial stress that adopting a healthy food program may place on management and staff, which, in turn, can affect staff morale, productivity, and retention.

## Best Practices for Production and Supply of Healthy Foods and Beverages

To address the overarching production and supply concerns, respondents considered the following to be best practices:

1. Regularly emphasize and share the goals of the healthy food program with your staff, emphasizing the benefits of a healthier diet.
2. Instill pride in the production of better tasting healthier foods, enhancing staff productivity and retention.
3. Conduct taste tests with frontline staff so they buy in to healthier options and will then market them to consumers.
4. Develop recipes that balance the cost of ingredients, but overall provide a healthy, delicious alternative.
5. Absorb losses associated with more expensive, healthier foods by offsetting higher costs with increases in the price of other popular dishes on the cafeteria’s larger menu.
6. Serve smaller portions of those foods that are more expensive to make.
7. Introduce innovative training methods that maximize the reach and impact of individual trainers.
8. Adopt innovative technologies and obtain kitchen equipment to enhance labor efficiency and productivity.
9. Work with your GPO to negotiate prices and secure reasonably priced ingredients for healthy foods.
10. When managing multiple sites or facilities, use POS systems to determine top-sellers and simplify inventory management.
11. Identify and work with small, local vendors to achieve healthy food goals.
12. Set up healthy stations to ensure portion control and distinguish healthy food choices.
13. Adopt behavioral design strategies to encourage healthy food selection and sales.

Six directors noted that implementing healthy food service guidelines is a journey that happens in incremental phases over time:

It’s a journey. It’s not going to happen overnight. We live in [western state], which is one of the healthiest states in the U.S., but people here want their [unhealthy food] as much as anybody else does and it’s hard to change people’s minds. So every little victory is a victory. (P01)

## The Journey Continues

Hospital food retail operations are increasingly pressured to provide healthier, but often more costly, food and beverage options to customers, while remaining financially sound. A perception that customers want less-healthy comfort foods on the menu has implications for food service directors attempting to offer healthy menus while maintaining profits. More research is needed on how best to incentivize and motivate customers to demand healthier options. Although we did not ask directly about subsidies, it is possible that hospitals that subsidize healthy menu items could have more success overall. Our study provides insights into how hospital food service directors can increase healthy options and maximize revenue and profit in their retail operations. Future research should examine quantitative sales data to determine how healthy foods and beverages perform financially when compared with less healthy menu items. The ultimate goal is to improve health and reduce disease risk among customers.
